# A Novel Homozygous *KIF1C* Variant in 2 Cases of Spastic Ataxia Type 2

**DOI:** 10.1212/NXG.0000000000200311

**Published:** 2025-10-16

**Authors:** Katariina Granath, Salla M. Kangas, Sanna Huhtaniska, Maria Suo-Palosaari, Veli-Pekka Ronkainen, Heli Helander, Elisa Rahikkala, Reetta Hinttala, Johanna Uusimaa, Jussi-Pekka Tolonen

**Affiliations:** 1 Department of Pediatrics and Adolescent Medicine, Division of Pediatric Neurology, Oulu University Hospital, Finland;; 2 Research Unit of Clinical Medicine, University of Oulu and Medical Research Center Oulu, Oulu University Hospital and University of Oulu, Finland;; 3 Department of Diagnostic Radiology, Physics and Technology, Research Unit of Health Sciences and Technology, and Medical Research Center Oulu, Oulu University Hospital and University of Oulu, Finland;; 4 Biocenter Oulu, University of Oulu, Finland;; 5 Department of Genomics, Turku University Hospital, University of Turku, Finland; and; 6 Department of Clinical Genetics, Oulu University Hospital, Finland.

## Abstract

**Objectives:**

Variants of unknown significance (VUS) pose an extensive clinical challenge. Our objective was to explore the diagnostic pipeline from symptom onset to molecular diagnosis in autosomal recessive (Spastic ataxia type 2 [SPAX2], Mendelian Inheritance in Man [MIM] number 611302) caused by a new homozygous variant in the *KIF1C* gene.

**Methods:**

Two unrelated individuals with early-onset spastic ataxia were evaluated for genetic etiology by exome sequencing. Case reports were compiled through a medical chart review. Two cellular models were established to assess variant pathogenicity.

**Results:**

Whole exome sequencing revealed a homozygous variant in *KIF1C* (NM_006612.6: c.833T > C, p.[Leu278Pro]) in a highly conserved motor domain of the KIF1C protein in both individuals. Two cellular models overexpressing a green fluorescent protein (GFP)-tagged KIF1C harboring the p.Leu278Pro variant demonstrated disrupted protein localization, suggesting an impaired trafficking capacity of the mutant KIF1C. A diagnosis of SPAX2 was established based on the in vitro data. Novel clinical findings associated with this *KIF1C* variant included retinal dysfunction detected by electroretinogram, hypotonia, and a thin corpus callosum in brain MRI.

**Discussion:**

Classification of pathogenicity requires extensive multidisciplinary effort, which can be burdensome for affected individuals and families. Like other proteins of the kinesin family, variants in KIF1C may underlie retinal dysfunction.

## Introduction

Next-generation sequencing (NGS) is helpful in diagnosing complex genetic disorders. However, NGS also identifies variants of unknown significance (VUS), which are a burden for affected patients and their families and clinicians.^1,2^

Spastic ataxia type 2 (SPAX2) is a rare neurologic disorder with a core phenotype of ataxia, dysarthria, and spasticity and symptom onset in the first 2 decades of life.^3^ SPAX2 is caused by pathogenic variants in *KIF1C*.^4-6^ The KIF1C protein is a microtubule-associated motor protein involved in various cellular processes, such as nervous system development, migration, and survival.^7-10^

Here, we report on 2 unrelated Finnish pediatric patients who share similar phenotypic presentations and a novel homozygous *KIF1C* variant, expanding the genotype-to-phenotype spectrum of *KIF1C*-related disease. We review our diagnostic pipeline in the context of SPAX2. Finally, we provide evidence for the pathogenicity of the KIF1C^Leu278Pro^ substitution through in vitro assays.

## Methods

### Study Setup

This study is part of the PEDIATAX project, a single-center study of early-onset cerebellar diseases. Informed consent was obtained from participating children and their guardians. The research protocol has been approved by the regional ethics committee (The Regional Medical Research Ethics Committee of the Wellbeing Services County of North Ostrobothnia; Eettinen Toimikunta [EETTMK]: 67/2019, amendments October 30, 2020; April 19, 2021; and December 19, 2024). The Declaration of Helsinki was followed. Highly confidential personal information is not published because ethical reasons. Clinical evaluations were performed by specialists in pediatric neurology, and patient charts were revisited to collect data. A literature cohort of previously published, genetically confirmed cases was retrieved from the PubMed database. The search terms included titles for the *KIF1C* gene and associated disorders (eAppendix 1).

### Variant Pathogenicity

Detailed methods are described in eAppendix 1. The *KIF1C* NM_006612.6: c.833T > C, p.(Leu278Pro) variant and its segregation was confirmed by Sanger sequencing. The pathogenicity of the variant was estimated using MobiDetails application programming interface (API)^11^ and Clustal Omega.^12^ To assess the pathogenicity in vitro, the GFP-tagged KIF1C^Leu278Pro^ protein was introduced in cellular models by transient transfection to quantify mRNA expression, protein abundance, and subcellular localization. All experiments were performed in biological triplicates.

## Results

### Clinical Data

**Figure 1:**
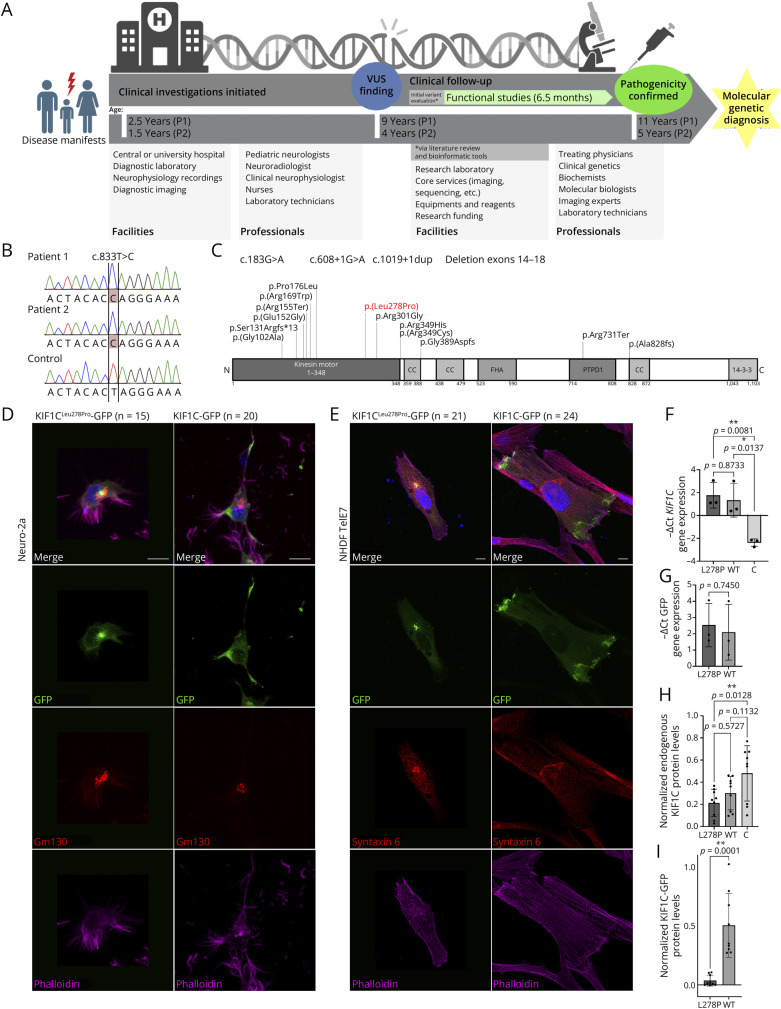
Multidisciplinary Team (MDT) Evaluation and Functional Modeling of KIF1C^Leu278Pro^ (A) The diagnostic pipeline from symptom onset to molecular diagnosis requires extensive facilities and an MDT of professionals to establish and confirm variant pathogenicity. (B) Sanger sequencing of blood-derived genomic DNA shows a homozygous NM_006612.6: c.833T > C in the 2 patient samples. (C) Schematic representation of the KIF1C protein domains with the new variant described in this study (red) and previously reported variants annotated to the current Matched Annotation from NCBI and EBI (MANE) select transcript. (D and E) Localization of overexpressed pKIF1C^Leu278Pro^-GFP or pKIF1C-GFP constructs in Neuro-2a cells and the immortalized human skin fibroblast NHDF line. The localization was analyzed through blinded cell scoring for 35 images of Neuro-2a cells (each captured field containing several eligible cells) and for n = 45 immortalized NHDF cells. The parenthetical number refers to the number of cells analyzed with each cell line and construct. Rows (up to down): Merge; GFP = overexpressed construct; Gm130 or Syntaxin 6 = Golgi apparatus; Phalloidin = actin filaments. Nuclei were counterstained with 4′,6-diamidino-2-phenylindole (DAPI) (blue). Scale bar = 10 μm. (F and G) *KIF1C* and GFP mRNA levels evaluated by quantitative PCR, normalized against housekeeping genes *GAPDH* and *TFRC*. (H and I) Protein abundance of endogenous KIF1C and overexpressed KIF1C-GFP by immunoblotting. All data were based on 3 independent experiments (mean ± SD). Statistical significance was assessed using analysis of variance (ANOVA) with a post hoc Tukey multiple comparisons test (≥3 groups) or the Student *t* test (2 groups). A *p* value of ≤0.05 was considered statistically significant.

The diagnostic pipeline from symptom onset to molecular diagnosis is depicted in Figure 1A, including the facilities and professionals required across the diagnostic odyssey. Exome sequencing revealed a homozygous substitution in *KIF1C* (NM_006612.6: c.833T > C, p.[Leu278Pro]) of a highly conserved leucine for both individuals, confirmed by Sanger sequencing (Figure 1B). The variant is ultra rare (total allele frequency of 0.000007 in gnomAD [v4.1.0, January 10, 2025]), only one heterozygous carrier being identified in the Finnish population.

**Figure 2:**
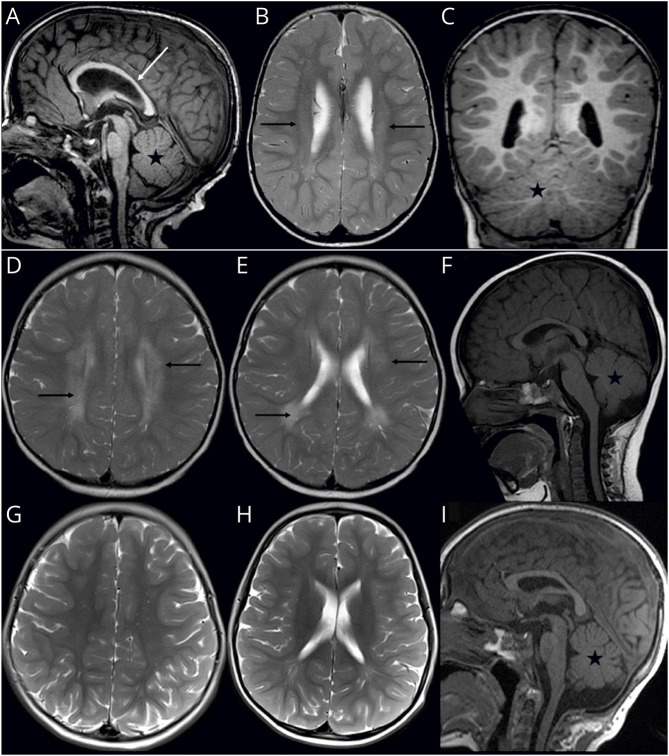
Brain MRI Findings Associated With KIF1C^Leu278Pro^ Patient 1 (A–C). Brain MRI at age 3 years and 2 months showed enlarged lateral ventricles, thin corpus callosum (A, white arrow), decreased amount of white matter, and mild symmetrical periventricular T2 signal hyperintensity (B, black arrows) presenting hypomyelination. Normal cerebellum (C, star) without pontocerebellar hypoplasia. Patient 2 (D–I). Brain MRI at age 1 year and 8 months showed periventricular T2-hyperintense white matter suggesting delayed myelination (D and E, black arrows), but at age 3 years and 8 months (G and H), myelination was normal. Normal cerebellum (F, I, star) without pontocerebellar hypoplasia.

See eAppendix 1 for detailed case reports. Brain imaging findings are presented in Figure 2. Patient 1 presented with ataxia, horizontal nystagmus, hypotonia, hyperreflexia, motor developmental delay, and visual impairment with retinal dysfunction. The presenting symptom was delayed motor development. MRI of the brain showed ventricular enlargement, thin corpus callosum, decreased amount of white matter, and hypomyelination. Patient 2 presented with spastic ataxia, epilepsy (GEFS+), mixed developmental disorder, intellectual disability, esophoria, and brisk tendon reflexes in lower extremities. The presenting symptom was the delayed motor development. MRI of the brain suggested delayed myelination.

**Figure 3:**
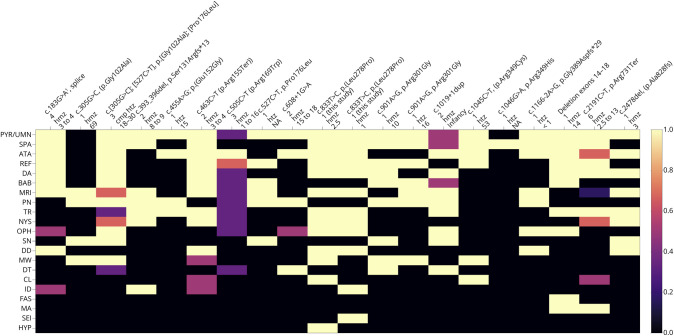
Genotypes-to-Phenotypes in Individuals With a Pathogenic *KIF1C* Variant The proportion of patients with the indicated phenotype per genotype is present as black (0) to light yellow (1.0). The variant, number of individuals with the indicated variant, zygosity, and the age at onset in years are provided in the column labels. References for the previously published individuals (n = 34) are provided in eAppendix 1. Footnote^1^: coding synonymous variant in the splice region—details in the eTable 1. ao = age at onset; ata = ataxia; bab = Babinski +; cl = clonus; da = dysarthria; dd = developmental delay; dt = dystonia; fas = fasciculations; hyp = hypotonia; id = intellectual disability; ma = muscle atrophy; mri = abnormality in brain MRI; mw = muscle weakness; nys = nystagmus; oph = ophthalmologicophthalmological signs; pn = peripheral neuropathy; pyr/umn = pyramidal / upper motor neuron signs; ref = hyperreflexia; sei = seizure; sn = sensory neuropathy; spa = spasticity; tr = tremor.

The literature search resulted in 34 genetically confirmed cases with 17 different disease-causing *KIF1C* variants (Figure 3) with very low or absent allele frequency in gnomAD (eTable 1). In 19 cases (56%), the variant was in the kinesin motor domain of the KIF1C protein (Figure 1C). Nine variants were potentially leading to loss of function (LOF), whereas 8 variants caused substitutions. All substitutions were in highly conserved residues and prediction tools tagged 6/8 deleterious (eAppendix 1, eAppendix 2). The age at onset ranged between 1 and 69 years (median = 8.5 years). The most frequently reported findings were upper motor neuron signs (n = 29%, 85%), including spasticity (n = 24%, 71%). Ataxia was reported in 26 cases (76%), with a severity of 10–30 on the Scale for the Assessment and Rating of Ataxia (SARA) scale. Abnormalities in brain imaging were reported in 19 cases (56%). No information was available on retinopathy, thin corpus callosum, or hypotonia in the literature. Functional in vitro data were available for 6 variants (eAppendix 2).

### KIF1C^Leu278Pro^ Function

We transfected mouse Neuro-2a and immortalized normal human dermal fibroblast (NHDF) cells with either the wildtype pKIF1C-GFP or the pKIF1C^Leu278Pro^-GFP construct. KIF1C^Leu278Pro^-GFP colocalized with the Golgi apparatus, while the wildtype protein gave the strongest signal from the cell periphery for all analyzed cells in both cell lines (Figure 1, D and E, and eFigures 1 and 2). The endogenous KIF1C displayed a typical expression pattern in Neuro-2a cells transfected with either of the 2 constructs (eFigure 3). In addition, *KIF1C* mRNA expression increased 4-fold in overexpressing cells in comparison with untransfected cells (*p* < 0.05, Figure 1F); the GFP mRNA levels were similar between the 2 KIF1C constructs (Figure 1G); the endogenous KIF1C protein levels decreased with pKIF1C^Leu278Pro^-GFP to 62% of those levels observed in untransfected control cells (*p* = 0.0128, Figure 1H and eFigure 4); and the pKIF1C^Leu278Pro^-GFP protein level was only 11% of the levels observed with pKIF1C-GFP (*p* = 0.0001, Figure 1I and eFigure 4). Transfection efficiency was greater with Neuro-2a than with NHDF cells.

## Discussion

This study describes our diagnostic pipeline from symptom onset to molecular diagnosis for a novel *KIF1C* variant (NM_006612.6: c.833T > C, p.[Leu278Pro]). We established 2 distinct cellular models to assess the functional impact of the variant in the kinesin motor domain, the most common location of disease-causing variants in *KIF1C*, confirming the diagnosis of SPAX2 for 2 unrelated individuals. Overall, the diagnostic odyssey has taken 4 and 8 years for these individuals, illustrating the extensive efforts required to evaluate a single VUS finding.

KIF1C is involved in the transportation of cargoes from the Golgi apparatus to the cell periphery.^7-10^ Our in vitro studies illustrated disrupted function for KIF1C^Leu278Pro^ through subcellular mislocalization. These data align with the cellular phenotype caused by KIF1C^Gly102Ala^, another disease-causing *KIF1C* variant in the motor domain with similar mislocalization in COS-7 cells.^4^ While evidence for a LOF effect for variants in the motor domain exists,^11^ the mechanism for most disease-causing *KIF1C* variants remain uncharacterized.^7^

In our experience, Neuro-2a cells were superior to immortalized NHDF cells in modeling KIF1C function because of higher transfection efficiencies. Of interest the wildtype pKIF1C-GFP and the pKIF1C^Leu278Pro^-GFP constructs overexpressed mRNA at similar levels, but the protein abundance of KIF1C^Leu278Pro^ was only 11% of the wildtype KIF1C. This finding could be explained by destabilization or increased degradation of the mutant protein^12^ or disrupted antibody binding (amino acid residues 1–273 vs variant at residue 278).

Novel phenotypic findings described in this study include KIF1C-associated retinal dysfunction, as one of our patients (P1) had a visual disability and retinal dysfunction based on electroretinogram. Although retinopathies have been linked to other members of the kinesin protein family (KIF1A^13^ and KIF11^14^), our literature review did not demonstrate similar co-occurrences with KIF1C. Our patient also presented with hypotonia and a thin corpus callosum, which were not described previously. For the rest, the phenotypic findings of the patients in this study aligned with the previously described phenotypes.

In conclusion, our findings expand the genotype-to-phenotype correlations of pathogenic *KIF1C* variants, with the first demonstration of *KIF1C*-associated retinal dysfunction. Limitations of this study are small sample size, although the high coverage of the PEDIATAX study enabled the identification of all eligible cases. The genomic Multidisciplinary Team approach is effective in increasing diagnostic yield, helping interpret and sometimes reclassify VUSs and improving patient management. Finally, our study highlights the challenges associated with VUS interpretation and the need for a systematic framework during the diagnostic odyssey.
